# Orally ingested *Lactobacillus crispatus* VPC177 enhances total vaginal *Lactobacillus* counts—a cross-over exploratory study

**DOI:** 10.3389/fmicb.2026.1894758

**Published:** 2026-09-03

**Authors:** Anna Berggren, Mary Farrell, Li Sun, Jessica Neilands

**Affiliations:** Probi AB, Lund, Sweden

**Keywords:** *Lactobacillus crispatus*, microbiota modulation, probiotics, vaginal health, vaginal microbiome

## Abstract

**Introduction:**

The vaginal microbiome plays a crucial role in vaginal health, and disturbances in the microbial balance can negatively affect female wellbeing. In recent years, there has been growing interest in the use of probiotics and prebiotics to support a healthy vaginal microbiota. However, effects are often strain-specific and should be clinically demonstrated to influence the vaginal microbiota. This study aimed at evaluating the ability of four different orally administered probiotic strains to affect the fecal, and more specifically, the vaginal microbiota in healthy women.

**Methods:**

In this open-label, cross-over, exploratory study 35 women age 20–63 years consumed four different strains, *Lactobacillus crispatus* VPC111, *Lactobacillus gasseri* VPG44, *L. crispatus* VPC177 and *Lactiplantibacillus plantarum* HEAL9^®^ for 14 days each with a washout period of 14 days between each intervention period. Levels of lactobacilli were assessed in fecal and vaginal samples following each intervention period.

**Results:**

VPC111, VPC177, and HEAL9 all resulted in significant increases in fecal lactobacilli after 14 days intervention compared to baseline (*p* < 0.05). VPC177 significantly increased vaginal lactobacilli (*p* = 0.001), whereas VPC111, VPG44, and HEAL9 showed no significant vaginal effects. *Post hoc* characterization of *L. crispatus* VPC177 demonstrated that it produces both D- and L-lactate at high levels (9.31 and 4.58 g/L, respectively) and possesses genes associated with bacteriocin production, glycogen utilization, and mucosal adhesion, supporting its potential for antimicrobial activity, carbohydrate metabolism, and colonization of the vaginal environment.

**Discussion:**

These findings demonstrate that probiotic effects on the vaginal microbiota are strain-specific, and that an increase in fecal lactobacilli does not necessarily translate into corresponding changes in the vaginal microbial community. Among the strains evaluated, VPC177 was the most promising candidate for supporting vaginal health through oral probiotic administration.

## 1 Introduction

Just as the intestinal microbiota influences gastrointestinal health, the vaginal microbiota, together with the vaginal epithelium, plays a vital role in maintaining the health and homeostasis of the female reproductive tract. In women of reproductive age, a healthy vaginal ecosystem is typically dominated by *Lactobacillus* species (Smith and Ravel, 2017). Lactobacilli promote a healthy vaginal ecosystem through several mechanisms including production of lactic acid to create an acidic environment, competitive exclusion of pathogens, as well as producing antimicrobial compounds such as hydrogen peroxide and bacteriocins (Chen et al., 2022). Researchers have categorized vaginal microbial compositions into five common community state types (CST), depending on which lactobacilli species is most dominant (Ravel et al., 2011). For example, CST type I is dominated by *Lactobacillus crispatus*, CST type II is dominated by *Lactobacillus gasseri*, CST type III by *Lactobacillus iners*, CST type IV is devoid of lactobacilli strains and CST type V is dominated by *Lactobacillus jensenii*. CST IV can be further subdivided depending on if any specific bacterial genus such as for example bifidobacteria is dominating.

Several intrinsic and extrinsic factors can cause disruptions to the vaginal microbiome such as antibiotic intake, immune status, different sexual partners, douching, diabetes, stress, and hormones (Borrego-Ruiz and Borrego, 2025). In particular, changes in estrogen levels can cause vaginal microbiome changes throughout a woman’s life (Romeo et al., 2024). During puberty, the increasing levels of estrogen thicken the vaginal epithelium and increase glycogen production, which when metabolized decreases the vaginal pH, favoring growth of lactobacilli (Smith and Ravel, 2017). Estrogen fluctuations during the menstrual cycle can affect the vaginal microbiota, influencing microbial diversity and *Lactobacillus* abundance (Kaur et al., 2020). During menopause the decreasing levels of estrogen, lead to a thinner vaginal epithelium, less glycogen production, and an increase in vaginal pH, resulting in a shift in microbial composition (Amabebe and Anumba, 2018; Chen et al., 2022; France et al., 2022; Gupta et al., 2019). Microbial dysbiosis in the vaginal microbiota have been associated with a range of urogenital conditions, such as bacterial vaginosis (BV), vulvovaginal candidiasis (VVC), urinary tract infections (UTIs), and increased susceptibility to sexually transmitted infections (STIs) (Lev-Sagie et al., 2022). Vaginal compositions with a decrease or lack of lactobacilli are associated with many of these adverse health outcomes. These less favorable microbial compositions can also influence reproductive outcomes such as fertility and pregnancy as well as the risk of pelvic inflammatory disease and preterm birth (Gupta et al., 2019). Current therapeutic strategies to modulate vaginal microbiota include hormonal therapy (estrogen), local treatment with lactic acid (to lower vaginal pH), and antibiotic treatment as a method to target urogenital pathogens but also to restore microbial balance (Abbe and Mitchell, 2023). The limitations of these therapeutic strategies including an increased risk of antibiotic resistance, disruption of commensal flora, and high recurrence rates, have prompted growing interest in probiotics as a potential preventive and therapeutic alternative (France et al., 2022; Gupta et al., 2019; Pagar et al., 2024).

The gut acts as a primary reservoir for many bacterial species that can also colonize the vagina (Amabebe and Anumba, 2018). These species originate from the gastrointestinal tract before reaching the vaginal niche through perineal transfer (Antonio et al., 2005). This suggests that orally administrated microorganisms that survive the gastrointestinal passage may appear in the vagina after a certain time. Optimal candidates for vaginal probiotic strains include commensal vaginal species, that can both colonize and exert health benefits by supporting a *Lactobacillus* dominated vaginal microbiota.

The aim of this exploratory study was to investigate the effect of three *Lactobacillus* and one *Lactiplantibacillus* strain on the fecal and more specifically vaginal microbiota following the oral intake of the lyophilized lactic acid bacteria as well as the tolerability of these strains in healthy females.

## 2 Materials and methods

### 2.1 Trial design

This was an open label, exploratory, four way cross-over study carried out at a single site at Probi AB, Lund, Sweden, between May 2005 and August 2005. The study consisted of telephone screening and eligible subjects came for an inclusion visit (Day 1) followed by eight subsequent visits (Day 15, 29, 43, 57, 71, 85, 99, and 113). After inclusion visit, four block periods were implemented consisting of a wash out period (WO) of 14 days and intervention period of 14 days, with each intervention period being subject to a different strain. Study assessments included fecal and vaginal sampling before each visit as well as collection of information on adverse events. The objective of this study was to investigate the presence of four different probiotic strains in fecal and vaginal samples following oral ingestion for 14 days and their effect on total lactobacilli counts. Before any study-related procedures, all subjects gave written informed consent, which specifically indicated that participation was voluntary and could be terminated at any time without any reason. The study was approved by Ethics Committee in Lund (protocol no. 928/2004) and conducted according to the principles expressed in the Declaration of Helsinki.

### 2.2 Study population

Healthy (self-reported) participants were recruited through the distribution of study information to employees at Ideon Medical Village in Lund and the Chemistry center at Lund University. The inclusion criteria were cisgender women aged 18–65 years. The exclusion criteria were employees of Probi AB, known intolerance or allergy to any ingredient included in the formulations, current treatment for severe gastrointestinal disorders and current treatment for vaginal disorders. Subjects who fulfilled the inclusion and exclusion criteria, were asked to participate in the clinical study and scheduled for an inclusion visit. Any intake of probiotics and/or antibiotics was prohibited, and each subject was provided with a list of probiotic products not allowed to be consumed during the study period. All concomitant medication was noted on the Case Report Form (CRF) and in the diary.

### 2.3 Sample size calculation

Although this was an exploratory study a sample size calculation was performed using statistical methods for rates and proportions. A study by Nobaek et al. (2000) demonstrated that *Lactiplantibacillus plantarum* 299v (DSM9843) was detected in fecal samples from 84% of the participants. It was assumed that the strains of this study would be found in fecal samples of fewer subjects and in even fewer subjects in samples from the vagina. The assumption was that the strains would be found in vaginal samples in 50%–60% of the participants. Including 30 subjects in the trial would therefore give maximum width of the 95% confidence interval for the proportion of subjects. The confidence interval width was calculated to be 0.3912 and precise enough to satisfy the scientific expectations. To adjust for potential dropouts 35 subjects were recruited.

### 2.4 Intervention

During the study, participants received four different strains, *L. crispatus* VPC 111 (DSMZ16741), *L. crispatus* VPC 177 (DSMZ16743), *L. gasseri* VPG 44 (DSMZ16737) and *L. plantarum* HEAL9 (DSM15312). The included strains had previously been tested in pilot clinical study showing promising results in being isolated from fecal and vaginal sample following ingestion of the strains in a suspended in an oat-milk/blueberry drink (Vásquez et al., 2005). Participants consumed two sachets of freeze-dried probiotic powder daily corresponding to 1 × 10^9^ CFU/day. From day 1 to day 113, the subjects were not allowed to ingest other products containing probiotic bacteria. Subjects who had not taken > 4 doses (2 days) in succession or had not taken a total of >8 doses (4 days) during each ingestion period of the study, had not followed the dietary regulations, or had started a course of medical treatment for severe gastrointestinal disease between day 1 and day 112 (on the discretion of the principal investigator) were excluded from the study as well as subjects who wished to discontinue the study.

### 2.5 Assessment of fecal and vaginal colonization

The ability of VPC111, VPC177, VPG44, and HEAL9 to colonize and affect the lactobacilli levels in the intestines and vagina was assessed by fecal and vaginal sampling. Samples were taken on the first day of each intervention period (Day 15, 43, 71, 99) as well as on the first day of the wash out (WO) period (Day 29, 57, 85, 113) following each intervention period. The sample on day 15, 43, 71, and 99 was collected before taking the first dose of the period’s study product. The fecal samples were collected using a fecal sampling kit no more than 18 h before being handed in for analysis, and during this period stored in a refrigerator. Vaginal samples were collected by inserting a vaginal swab covered by a protective cap into the vagina, with the cap reducing the risk of bacterial contamination during insertion/collection (Copan amies agar gel swabs; Copan innovation, Brescia, Italy). The samples were collected no more than 3 h before being handed in for analysis, and during this period stored in a refrigerator.

### 2.6 Analysis of fecal and vaginal samples

The fecal samples were weighed before being dissolved in 9 ml of sterile phosphate-buffered saline (PBS), pH 7.2 and serially diluted while the vaginal swabs were agitated in 9 ml PBS prior to serial dilution. Aliquots of each dilution of the vaginal sample and the last three dilutions of the fecal samples were plated on Rogosa agar (Oxoid AB, Sollentuna, Sweden) and anaerobically incubated for 3 days in a BBL Gas Pack Anaerobic System (Becton Dickinson Microbiology Systems, Cockeysville, MD, United States) at 37 °C. The samples were analyzed based on colony morphology and number of colony forming units (CFU) of each morphology according to Johansson et al. (1998). Detection limit for both total lactobacilli counts and the intervention strains was log 3.0 for fecal samples and log 2.0 for vaginal samples. Adjustments for differences in stool weight were made.

### 2.7 Randomly amplified polymorphic DNA (RAPD)

Presence of the administered study strains was identified with RAPD according to Johansson et al. (1995). Briefly, representative colonies of the different morphologies on Rogosa agar were isolated and purified on Rogosa agar and stored in freezing buffer at −80 °C until further analysis. Isolates were cultivated overnight in *Lactobacillus* carrying medium (LMC) (Efthymiou and Hansen, 1962) supplemented with 1% glucose. Cells from 100 ml cells suspension were collected by centrifugation and washed twice in 1 ml sterile distilled water. The cells were resuspended in 0.25 ml distilled water and disintegrated by vigorous shaking with glass bead (0.2 mm diameter) using IKA Vibrax VXR shaker (IKA Labortechnik Staufen, Germany) at the maximum speed for 30 min in the cold. The disrupted cells were pelleted by centrifugation and 1 ml of the supernatant was used for the PCR. The PCR was carried out in a DNA Thermal Cycler 480 (Perkin Elmer, Norwalk, United States) using 9-mer primer (ACGCGCCCT; Pharmacia Biotech, Sweden) at a concentration of 8 μmol l^–1^. The nucleotides were added at a concentration of 0.2 mmol l^–1^ of each dNTP (Perking-Elmer, Barnchburg, NJ, United States), THE Taq-polymerase concentration was 50U ml^–1^ (Boehringer-Mannheim) and the concentration of MgCl was 1.5mmol l^–1^ (10xPCR buffer Boehringer Mannheim). The reaction mix was overlaid with mineral oil (Perkin-Elmer) and the temperature profile described by Johansson et al. (1995) was applied. The gel electrophoresis was carried out as described previously described and the DNA fragment profiles of the isolates were compared visually with the fragment profile of the investigated strain that was run as control on all gels (Johansson et al., 1995).

### 2.8 D/L lactate production

The D-/L-lactate was analyzed with D-/L-Lactic Acid (D-/L-Lactate) (Rapid) Assay Kit (Megazyme/Neogen, SKU 700004276, reference K-DLATE) following manufacturer instructions.

### 2.9 Bioinformatics

A curated bacteriocin reference database was constructed using the UniProt Knowledgebase (UniProtKB) (The UniProt Consortium, 2025). Protein sequences were retrieved for bacteriocin, mucus binding proteins and S-layer proteins. Fasta files were downloaded from UniProt on 22 April 2026 and formatted as local BLAST protein databases using makeblastdb (BLAST+) (Camacho et al., 2009).

Protein sequence similarity searches were performed using BLASTp against the respective local databases. For bacteriocins, hits were considered relevant when sequence identity exceeded 30% and query coverage was greater than 50%. For mucus binding proteins and S-layer proteins, more stringent criteria were applied to reduce false positives arising from low complexity or repeat regions: hits were retained when sequence identity exceeded 50%, query coverage was greater than 70%, and the annotation was consistent with known surface associated adhesins or S-layer proteins. Redundant hits to the same query locus were collapsed, and final annotations were assigned based on the best scoring reference sequence and domain composition.

### 2.10 Safety and tolerability

Safety and tolerability were assessed via monitoring of adverse events (AEs).

### 2.11 Statistical analysis

Statistical analysis was performed using SPSS (version 29) using non-parametric tests (Wilcoxon-Match Signed Ranked test) comparing the median values before and after intake of the investigational product. Level of significance was set at *p* ≤ 0.05 but since this was an exploratory study, some *p*-values at > 0.05 were also reported. *Post hoc* analysis was carried out on Graph Pad Prism to compare median values for total lactobacilli at end of administration for each bacterial strain compared to VPC 177 using the Wilcoxon rank sum test.

## 3 Results

### 3.1 Study population

**FIGURE 1:**
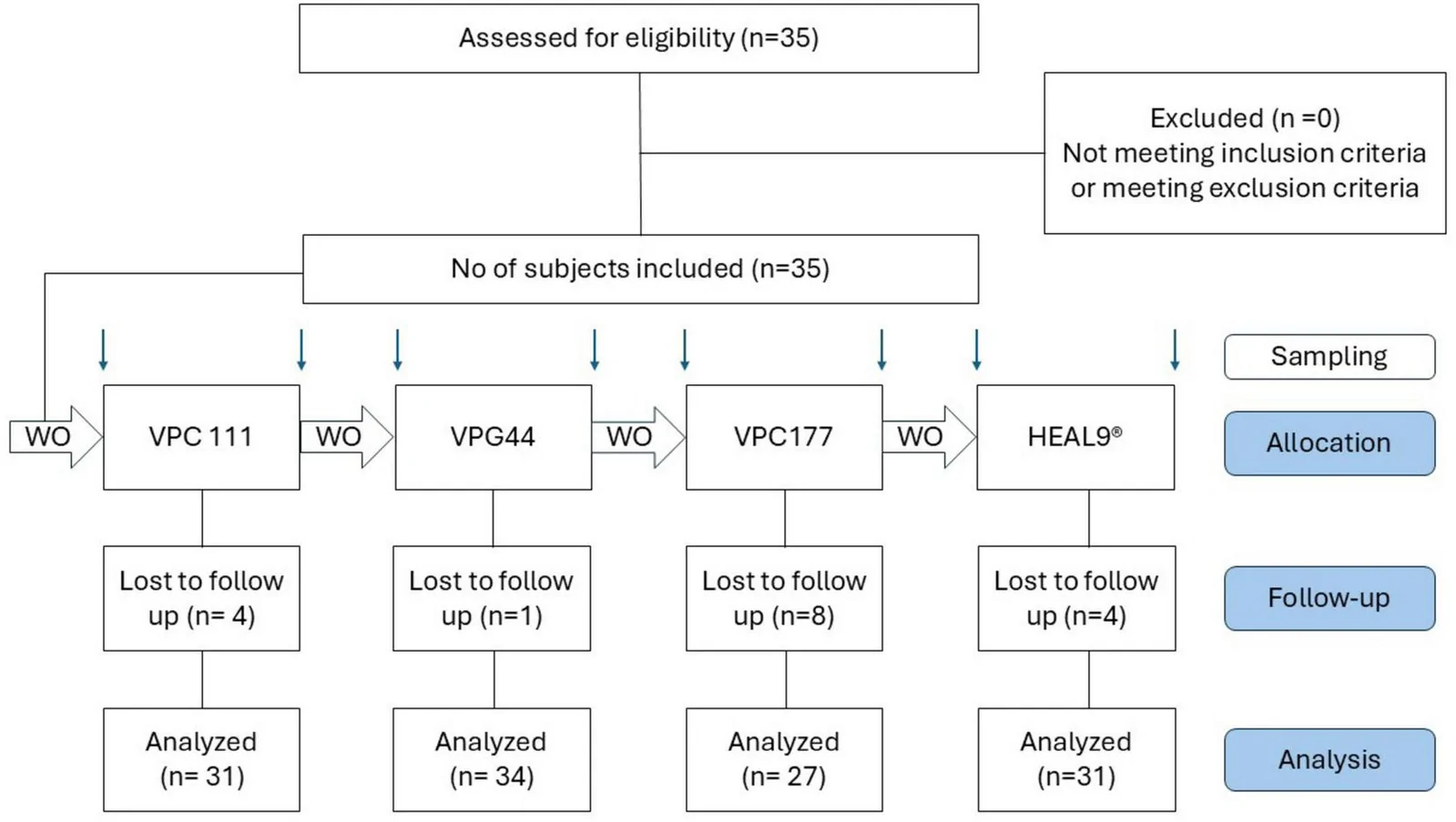
Modified CONSORT flowchart of the study.

The age of the participants ranged from 20 to 63 years of age with a median age of 33 years. Of the 35 women enrolled in the study, 31 completed periods 1 and 2 (washout + VPC111), 34 completed periods 3 and 4 (washout + VPG44), 27 completed periods 5 and 6 (washout + VPC177), and 31 completed periods 7 and 8 (washout + HEAL9). A total of 25 participants completed all study periods (Figure 1).

### 3.2 Lactobacilli count in fecal samples

**FIGURE 2:**
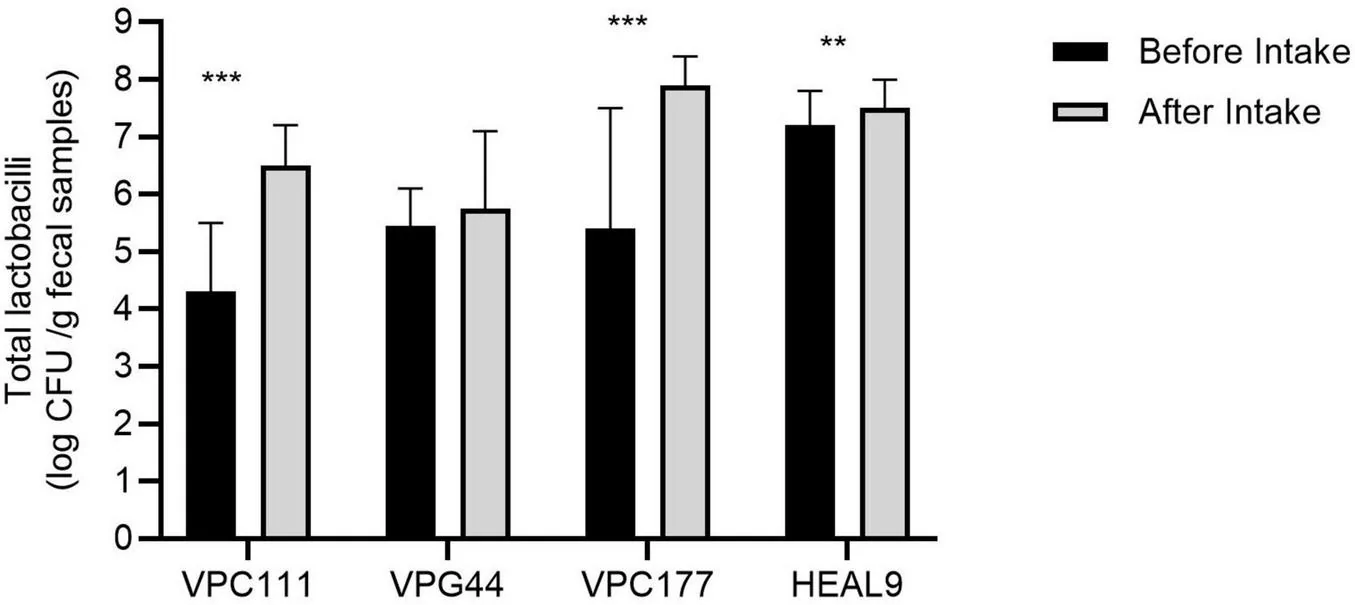
Total lactobacilli log CFU/g of fecal samples before and after 14 days consumption of VPC 111, VPG 44, VPC 177, and HEAL9. Results presented as median and 95% confidence interval. **p < 0.01, ***p < 0.001 for within group comparison (baseline vs. end of intervention, 14 days) using Wilcoxon-Match Signed Ranked test.

Three of the administered strains, VPC111, VPC177, and HEAL9 induced a statistically significant increase in total fecal lactobacilli counts (Figure 2). Specifically, VPC111 and VPC177 both resulted in a 1.7 log increase (*p* < 0.001), while HEAL9 led to a 0.8 log increase (*p* = 0.006). Although VPG44 showed a trend toward increased lactobacilli levels (0.7 log), the effect did not reach statistical significance (*p* = 0.071) (Figure 2).

Analysis of the presence of the administered strains using RADP analysis revealed that HEAL9 was below the detection limit (<log 3.0) in the sample taken before intervention but demonstrated a significant increase in fecal abundance following administration (*p* < 0.0001), with the strain being above detection limit in 27 out of 31 subjects after intervention. VPG44 was detected in one subject prior to administration and in four subjects following intervention but this increase was not significant (*p* = 0.125). For both VPC177 and VPC111, the administered strains were detected in a single subject pre-intervention but were not detected in the post-intervention samples. Looking at participants who provided fecal samples for all bacterial strains, *n* = 22, VPC177 resulted in significantly higher total amount of lactobacilli following intake compared to VPC111 (*p* = 0.0057) and VPG44 (*p* = 0.037), but not HEAL9 (*p* = 0.92).

### 3.3 Lactobacilli count in vaginal samples

**FIGURE 3:**
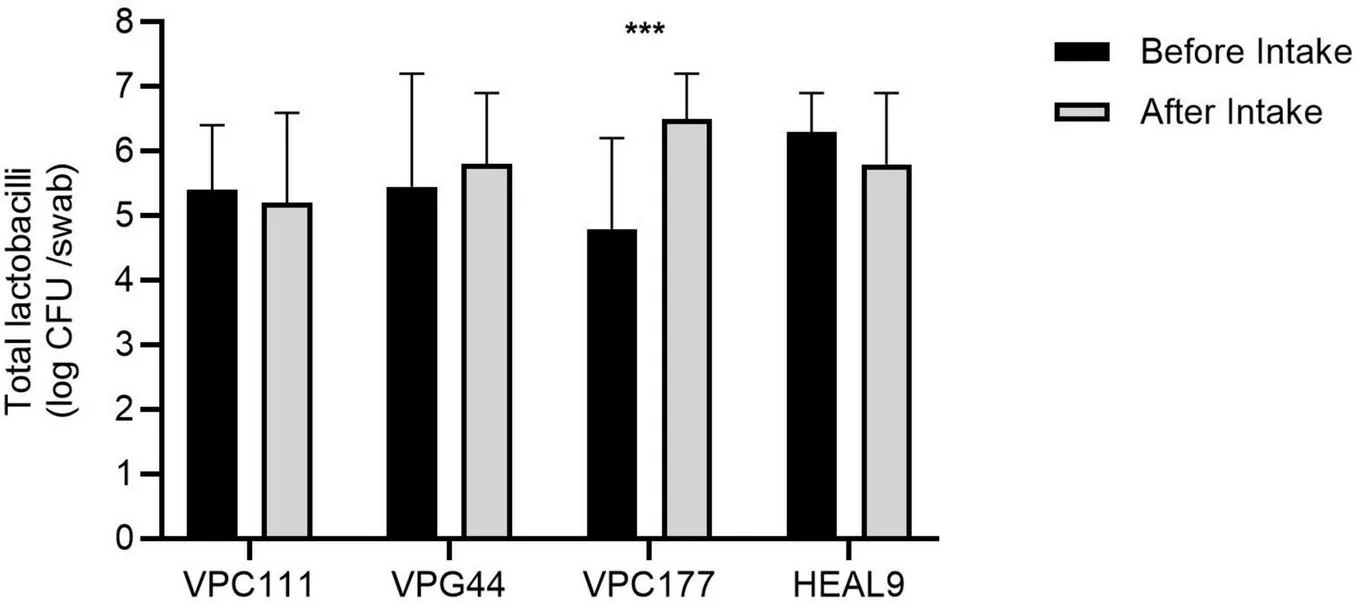
Total lactobacilli log CFU/swab of vaginal fluid samples before and after 14 days consumption of VPC 111, VPG 44, VPC 177, and HEAL9. Results presented as median and 95% confidence interval. ***p < 0.001 for within group comparison (baseline vs. end of intervention, 14 days) using Wilcoxon-Match Signed Ranked test.

Quantification of total lactobacilli in vaginal samples revealed a significant increase associated with only one strain, VPC177, which elevated the median count from 4.9 log to 6.5 log (*p* < 0.001) and also displayed the highest total median value of the four different strains (Figure 3). Analysis of the presence of the administered strains in vaginal samples showed that VPC177 was detected in two subjects prior to intervention as well as post-intervention, while in the remaining subjects it was below detection limit. Both VPC111 and HEAL9 were below detection limit prior to intervention; however, VPC111 was detected in three subjects and HEAL9 in one subject following administration. The VPG44 strain was below detection limit both before and after the intervention.

When looking at participants who provided vaginal samples for all bacterial strains (*n* = 25), VPC177 resulted in significantly higher total amount of lactobacilli following intake compared to VPC111 (*p* = 0.0065), VPG44 (*p* = 0.003), and HEAL9 (*p* = 0.009).

### 3.4 Bacterial properties of *L. crispatus* VPC177 important for vaginal colonization

Lactic acid production was measured using a lactic acid kit. VPC177 produced both D- and L-lactate (9.31 and 4.58 g/L, respectively). To further characterize VPC177 genome analysis of antimicrobial metabolite production, glycogen utilization, and mucosal adhesion was conducted. Sequence similarity searches against the curated bacteriocin reference database identified three loci with homology to bacteriocin associated proteins (Supplementary Table 1). The locus NBEEHJGG_01789 showed 57.5% amino acid identity to a helveticin J–like bacteriocin. In addition, two loci, NBEEHJGG_02018 and NBEEHJGG_02060, showed 100% amino acid identity and full query coverage to bacteriocin-type signal sequence peptides from *L. crispatus*. BLASTP analysis revealed that NBEEHJGG_00190 encodes a protein showed 99.3%–100% amino acid identity and 100% query coverage to annotated pullulanase (pulA) proteins from *L. crispatus* reference strains, indicating a potential role in glycogen or complex carbohydrate utilization.

Two mucus binding protein genes were identified in the genome, including one canonical mucus binding protein (627 aa) and one MBG domain containing surface protein (534 aa) (Supplementary Table 2). Both showed moderate sequence identity to reference lactobacilli proteins, consistent with the known diversity and host-adaptation of mucus binding adhesins. Thirteen high confidence S-layer associated proteins were identified, including multiple canonical S-layer proteins and SLAP domain containing anchoring proteins. All retained candidates showed ≥97% amino acid identity and full-length coverage to *L. crispatus* reference sequences (Supplementary Table 3).

### 3.5 Safety and tolerability

The study products were well-tolerated. One female reported a fungus infection while consuming VPC111 and four individuals reported mild gastrointestinal issues while ingesting VPG44. No adverse events were reported with VPC177 nor HEAL9 intake.

## 4 Discussion

The normal vaginal microbiota is believed to originate from the rectal mucosa and perineal area, suggesting that orally administered probiotic strains capable of surviving gastrointestinal transit may eventually colonize the vaginal environment (Amabebe and Anumba, 2018). This study aimed to evaluate the capacity of four orally administered lactic acid bacteria to colonize and affect the gastrointestinal and more specifically the vaginal microbiota in healthy women. The results of this study demonstrate that oral administration of specific strains can modulate the fecal but, to a lesser extent, the vaginal lactobacilli population in healthy women. The findings support the concept of a gut-vagina axis, although the extent of this effect appears to be strain-specific and limited compared with the impact on the gut microbiota.

Three of the four tested strains, VPC111, VPC177, and HEAL9, elicited a statistically significant increase in total lactobacilli counts in fecal samples, indicating their capacity to influence the gut microbial composition. VPC111 and VPC177 both resulted in a substantial increase, while HEAL9 led to a more modest yet statistically significant rise compared to baseline. Of the four administered strains only HEAL9 was above the detection threshold and isolated in 27 of 31 subjects following administration, confirming successful passage and at least transient establishment in the gut to detectable numbers. The limited detection of VPC111 and VPC177 post-intervention, despite increases in total lactobacilli, may indicate that these strains, although not being numerically dominant, can exert effects indirectly, potentially by stimulating growth of indigenous lactobacilli. VPG44 did not induce a statistically significant increase in fecal lactobacilli, although there was a trend toward significance. This may reflect lower colonization capacity and strain-specific differences in gastrointestinal survival (Colodner et al., 2003). Tolerance to bile acids is an important feature of probiotic strains for survival in the gastrointestinal tract (Ruiz et al., 2013). VPG44 exhibits substantially lower bile tolerance than the other strains (data not shown), which may have impaired its ability to colonize the gut, whereas the other three strains display high bile tolerance.

In the vaginal samples, only VPC177 was associated with a significant increase in total lactobacilli levels, suggesting a potential link between oral administration and modulation of the vaginal microbiota for this strain. The data from this study suggests that regardless of colonizing the vagina in detectable numbers, orally administered lactobacilli can still influence the vaginal ecosystem which is in line with previous studies (Macklaim et al., 2015; Koirala et al., 2020). Comparative genomics for other *L. crispatus* strains has also confirmed that *L. crispatus* naturally occurs in both human fecal and vaginal samples, underscoring its inherent cross−niche adaptability (Zhang et al., 2020). Intestinal colonization of lactobacilli has been suggested to be relevant for effect on the vaginal microbiota, as the gastrointestinal tract is considered a key reservoir for lactobacilli that can subsequently populate the vaginal environment, thereby contributing to the maintenance of vaginal eubiosis (Petricevic et al., 2013). However, this study indicates that a strain’s ability to colonize the gut and increase gut lactobacilli levels does not necessarily translate into effects in the vaginal microbiota. This is illustrated by the high abundance of gut lactobacilli and detections rates of HEAL9 following administration without a corresponding increase in vaginal lactobacilli, whereas for VPC177 the strain was not detected in the fecal samples but still induced a change in vaginal *Lactobacillus* counts.

The observation that an orally ingested *L. crispatus* increases total lactobacilli counts in the vagina is consistent with previous studies showing that *L. crispatus* strains can colonize the gastrointestinal tract and subsequently be detected in the vagina, contributing to shifts toward *Lactobacillus*-dominated vaginal microbiota. (Strus et al., 2012; Bertuccioli et al., 2022; de Leo et al., 2023; Filardo et al., 2024). In healthy women with intermediate flora a 60-day consumption of *L. gasseri* 57C, *L. fermentum* 57A, and *L. plantarum* 57B resulted in an increase total number of lactobacilli in the vagina (Strus et al., 2012). Macklaim et al. (2015) investigated the effect of tinidazole plus *Limosilactobacillus reuteri* RC-14 and *Lactobacillus rhamnosus* GR-1 in women with BV and showed that tinidazole alone did not affect the vaginal microbiota while the addition of orally ingested probiotics increased the number of lactobacilli in the vaginal samples. Similar to this study, the administered strains were not detected in the vaginal samples. The ecological concept of keystone species suggest that species of low abundance may have effects on microbial communities that are disproportionate to their abundance (Banerjee et al., 2018; Han et al., 2022; Han and Vaishnava, 2023). This concept could potentially partly explain why some species elicit an effect on the vaginal microbiota without being numerically dominant or even detected themselves as in the case of VPC177 and the strains in the study by Strus et al. (2012), Macklaim et al. (2015). Reports on vaginal colonization by *Lactobacillus* strains have been inconsistent, emphasizing that this trait is strain dependent. For example, de Leo et al. (2023) demonstrated that *L. crispatus* NTCVAG04 can reach the vaginal niche following oral administration, this was also the true for *L. gasseri* CECT 30648 but not *L. crispatus* CECT 30647 in the same study (Perez et al., 2025). On the contrary Hertz et al. (2022), Qi et al. (2023) reported that *L. gasseri* DSM 14869, *L. gasseri* TM13, and *L. crispatus* LG55 fail to colonize the vaginal environment. Comparable observations have been documented for strains belonging to other *Lactobacillus* species (Russo et al., 2018; Koirala et al., 2020; Zhang et al., 2021), collectively reinforcing that successful transit along the oral-gut-vaginal axis represents a selective process achievable by certain well-adapted strains.

Another possible explanation for the different effects on the vaginal microbiota seen in this study could lie in differences in immunomodulatory properties. Recent data indicates that different strains of vaginal commensals such as lactobacilli interact with TLR and anti-inflammatory receptors to differing degrees and could therefore result different immune responses which potentially could impact microbial composition in the vagina (Decout et al., 2024). While the primary immunomodulatory effects are observed in the gastrointestinal tract, emerging evidence suggests that modulation of gut mucosal immunity may also influence immune responses at distal mucosal sites, including the female reproductive tract (Gill et al., 2010). Through mechanisms associated with the common mucosal immune system, gut-derived immune signals may contribute to the regulation of vaginal immune homeostasis. A less inflammatory vaginal environment may favor the growth and persistence of protective *Lactobacillus* species (Agoni et al., 2026; Landolt et al., 2025). Although the mechanistic links between gut immune modulation and vaginal microbiota composition remain incompletely understood, these observations could support that gut-targeted interventions could indirectly support a *Lactobacillus* dominated vaginal ecosystem as seen in this study. However further studies will be needed to investigate the immunomodulatory properties of VPC177 in relation to impact on the vaginal microbiota.

Other key properties of lactobacilli important for vaginal colonization and microbial modulation includes ability to adhere to the epithelial lining of the vaginal mucosa, ability to degrade and ferment glycogen into predominantly lactic acid, which help sustain an acidic pH (typically between 3.8 and 4.2) creating conditions that inhibit the growth of pathogenic microorganisms, production of antimicrobial peptides (AMPs) and hydrogen peroxide both of which exhibit direct antibacterial and antifungal properties, effectively suppressing a wide range of pathogens (Chen et al., 2022). A characterization of VPC177 based on these important properties was conducted. *L. crispatus* strains typically produce several grams per liter of lactic acid under laboratory conditions, with reported values around ∼9 g/L total lactate. *In vivo*, lactate concentrations in *Lactobacillus*-dominated vaginal environments with Nugent score 0–3, are approximately ∼10 g/L (Tachedjian et al., 2017). Therefore, the production levels of VPC177 approaching 10 g/L can be considered indicative of strong lactic acid production. Genome analysis indicated the presence of pullulanase (pulA). The N-terminal of this gene sequence was identical to the strains with high-efficiency growth on glycogen (van der Veer et al., 2019). Glycogen degrading ability was also confirmed using an API50 CHL test (data not shown). As far as ability to adhere to the vaginal mucosa the bioinformatics analysis revealed the presence of thirteen S-layer associated proteins and two mucus binding proteins in VPC177 indicating a highly developed and conserved surface layer architecture. For antimicrobial metabolites, despite the presence of a helveticin-family bacteriocin homolog, analysis of the ±10 kb genomic region surrounding NBEEHJGG_01789 revealed no co-localized genes encoding a dedicated ABC transporter, immunity protein, or other bacteriocin specific accessory functions. Instead, the gene was embedded within a region predominantly associated with metabolic functions and was flanked by a mobile genetic element. This genomic organization suggests that NBEEHJGG_01789 represents an orphan or cryptic bacteriocin like gene, rather than part of a complete and functional bacteriocin operon.

A notable limitation of this study lies in the methodology employed for detecting the administered strains. Specifically, identification was performed using Random Amplified Polymorphic DNA (RAPD) analysis following the isolation of dominant colonies on Rogosa agar through serial dilution. This approach carries the inherent risk that less abundant species may be masked by more dominant strains, potentially leading to their underrepresentation or omission in higher dilution samples. In addition, *L. crispatus* and *L. gasseri* are more oxygen sensitive than *L. plantarum* which could have affected the ability to cultivate them and therefore affected the RAPD results. In addition, the small sample size and the lack of placebo control are limitations and should be addressed in follow-up studies. Another limitation of the study was that the community state type (CST) of the participants was not assessed before each intervention period. Although the women served as their own controls, potential shifts toward vaginal dysbiosis between intervention periods may have influenced the ability of the strains to affect vaginal lactobacilli abundance.

Taken together, the results from this study suggests that not all strains exhibit equivalent colonization capacity or influence on host microbiota. VPC177 appears promising, demonstrating effects in both gut and vaginal compartments with bacterial properties that could support vaginal compatibility. However, the exploratory nature of this study warrants further research to clarify the mechanisms underlying the strain specific effect of VPC177 and to further assess efficacy of VPC177 in randomized placebo-controlled studies.

## Data Availability

The original contributions presented in this study are included in the article/Supplementary material, further inquiries can be directed to the corresponding author.
